# Association Between Access to Health Information and Frailty in Older Japanese Adults: Web-Based Cross-Sectional Study

**DOI:** 10.2196/83642

**Published:** 2026-02-27

**Authors:** Noriko Hori, Jiaqi Li, Yosuke Osuka

**Affiliations:** 1 Department of Frailty Research, Research Institute, Center for Gerontology and Social Science, National Center for Geriatrics and Gerontology, 7-430 Morioka-cho, Obu City, 474-8511, Japan, 81 0562-46-2311 ext 5261; 2 Department of Public Health, Faculty of Medicine, Kindai University, Sakai, Japan

**Keywords:** health literacy, frailty, internet, web-based survey, health information sources

## Abstract

**Background:**

Older adults often access traditional media, such as newspapers, magazines, television, and radio, for health information. However, compared with older adults without frailty, older adults with frailty experience greater declines in physical functions and mental health (including depressive symptoms), as well as social functioning, due to reduced interaction with others, which limits their access to these sources of information.

**Objective:**

This study aimed to identify the health information sources that are less accessible to participants with frailty than to those without frailty.

**Methods:**

A cross-sectional web-based survey was conducted among independent Japanese adults aged ≥75 years. We assessed frailty using the Questionnaire of Medical Checkup for Old-Old, with a score of ≥4 indicating frailty. Participants were asked whether they had accessed any health information source in the past year, including medical institutions, family members, friends or acquaintances, neighbors, government agencies, long-term care or welfare services, television, radio, the internet, magazines, newspapers, or books. The primary explanatory variable was frailty status. Covariates included age, sex, income, education, living arrangements, and health literacy, measured using the eHealth Literacy Scale.

**Results:**

In total, 1032 participants (n=518, 50.2% male; median age: 77 y) were analyzed. Multivariable logistic regression analysis revealed that participants with frailty had significantly less access to the following sources of information compared to individuals without frailty: family (odds ratio [OR] 0.69, 95% CI 0.50‐0.95), friends/acquaintances (OR 0.70, 95% CI 0.51‐0.98), radio (OR 0.50, 95% CI 0.31‐0.79), and newspapers (OR 0.66, 95% CI 0.50‐0.88). Sex-based subgroup analyses revealed no significant interaction effects, indicating no heterogeneity in the findings.

**Conclusions:**

Older adults with frailty were less likely to obtain health information from interpersonal and traditional media sources than did individuals without frailty. Health information providers need to devise strategies for delivering accurate information and improving usability to enable older adults with frailty to proactively access diverse health information.

## Introduction

Access to reliable health information is essential for individuals to recognize health concerns and engage in appropriate health management behaviors [1-3]. Among community-dwelling older adults, traditional media such as newspapers, magazines, television, and radio remain the predominant sources of health information, while interpersonal sources also play a meaningful role [3-5]. Greater access to diverse information sources has been associated with better health status and improved problem-solving abilities regarding health issues [6], highlighting the importance of understanding disparities in access among older adult populations.

Frailty is a clinical geriatric condition characterized by diminished physiological reserves and reduced resilience to stressors, contributing to a heightened risk of disability and adverse outcomes [7]. In Japan, the prevalence of frailty among adults aged ≥65 years is estimated at 7.4%, increasing sharply with age: 10% at ages 75‐79, 20.4% at ages 80‐84, and 35.1% at ages ≥85 [8]. Although frailty is potentially reversible, timely access to accurate and actionable health information may play a vital role in preventing severe deterioration, facilitating early access to support services, and maintaining health-related behaviors.

Older adults with frailty often experience reduced physical, cognitive, and social functioning compared with individuals without frailty [9-11], and declines in vision and hearing have also been linked to frailty [12-15]. These limitations may pose substantial barriers to accessing both interpersonal sources and traditional media, potentially restricting opportunities to obtain essential health information. Prior research has indicated that insufficient social support is associated with the progression of frailty and that access to multiple information sources contributes to healthier behaviors [6,16]. However, it remains unclear which specific types of information sources older adults with frailty are less likely to access. Addressing this knowledge gap is crucial for public health informatics, as targeted communication strategies must be grounded in an accurate understanding of how frailty affects information acquisition.

Therefore, this study aimed to compare access to health information sources between community-dwelling older adults with and without frailty and to identify specific sources that may be less accessible among those with frailty. Findings from this study may inform the development of tailored health communication strategies and interventions to reduce information disparities among vulnerable older populations.

## Methods

### Study Design and Setting

This study was a cross-sectional internet-based survey conducted over 2 days, from June 12 to 13, 2024.

### Participants

Participants were recruited through a panel management company affiliated with Cross Marketing Inc, a Japanese internet research firm. Approximately 50,000 individuals aged ≥75 years were registered as potential respondents to this survey. This study was designed to collect data from 1000 male and female participants across Japan, aged ≥75 years, who were certified as not requiring long-term care. Notably, 10,807 panel members aged ≥75 years were identified as potential respondents. Each panelist could log onto a secure website using a unique ID and password. Recruitment was conducted through this protected site, and interested individuals were asked to read and complete an online informed consent form. Only those who agreed to participate were allowed to complete the questionnaire. Responses were recorded only if all survey items were completed.

### Eligibility Criteria

Inclusion criteria were as follows: (1) age ≥75 years, (2) not certified as requiring support or long-term care under the national long-term care insurance system, and (3) provision of informed consent after reviewing the study information. Participant recruitment was terminated when responses exceeded 1000 eligible individuals.

### Outcome Measures

Based on previous studies [17-19], the primary outcomes were defined as the use or nonuse (0=not used, 1=used) of 12 health information sources over the past year: health care providers, family members, friends/acquaintances, neighbors, government agencies, long-term care and welfare professionals, television, radio, internet, newspapers, magazines, and books.

### Frailty Assessment

Frailty was assessed using the Questionnaire of Medical Checkup for Old-Old (QMCOO) [20], a 15-item screening tool that evaluates domains such as physical function, physical activity and falls, cognitive function, and social participation. Since 2020, the QMCOO has been widely implemented as part of health checkup questionnaires for individuals aged ≥75 years across municipalities in Japan. Moreover, it has demonstrated construct validity for frailty assessment [20], criterion-related validity [21], and predictive validity for mortality and new long-term care certification [22]. Table S1 in Multimedia Appendix 1 lists the items and response options for the QMCOO. Responses indicating poorer health or function were scored 1 point, whereas favorable responses were scored 0 points. These answers are used to calculate points for each question, and the scores range from 0 to 15. A total score of ≥4 points was classified as frailty [21,23]. For the analysis, frailty was treated as a binary variable (0=nonfrail, 1=frail).

### Covariates

Covariates were selected with reference to prior studies and included age, sex, final educational attainment level, household income, marital status, the presence of children, living alone [4,19,24], and health literacy. The final educational attainment level was classified into four categories: up to high school, technical or junior college, university or graduate school, and other. Household income was divided into six categories [25]: <2 million yen, 2 to <3 million yen, 3 to <4 million yen, 4 to <5 million yen, ≥5 million yen, and “don’t know/prefer not to answer” (1 million yen=US $6460).

Health literacy was measured using the 8-item eHealth Literacy Scale (eHEALS) [26], presented in Table S2 in Multimedia Appendix 1. Each item was rated on a 5-point Likert scale (1=strongly disagree to 5=strongly agree; total score range: 8‐40). Following prior research, total scores were dichotomized at the median (0=high, 1=low).

### Statistical Analysis

Categorical and continuous variables were summarized as percentages and medians with interquartile ranges, respectively. To examine the association between frailty and each health information source, a multivariable logistic regression analysis was conducted.

After confirming the absence of multicollinearity, the models were specified as follows.

The dependent variable was the use (0) or nonuse (1) of each source, the explanatory variable was frailty, and the covariates included health literacy and demographic variables. All covariates were entered simultaneously into the model.

To assess the robustness of the findings, subgroup analyses based on sex were performed. Subsequently, the interaction terms were tested for statistical significance.

All analyses excluded individuals who discontinued the survey or had missing responses. The survey company also automatically excluded fraudulent responses based on the following criteria: (1) extremely short response times suggesting that items were not read and (2) identical response options recorded for all items.

All analyses were performed using SPSS (version 29; IBM Japan) with a two-sided significance level of 5%.

### Ethical Considerations

This study was conducted in accordance with the principles of the Declaration of Helsinki and approved by the Conflict of Interest and Ethics Committee of the National Center for Geriatrics and Gerontology (approval number 1800). Informed consent was obtained from all participants via a web-based platform. The survey company provided deidentified data. As compensation, participants received points equivalent to several tens to several hundred Japanese yen, according to the survey company’s incentive policy.

## Results

### Participant Enrollment and Characteristics

**Figure 1.**
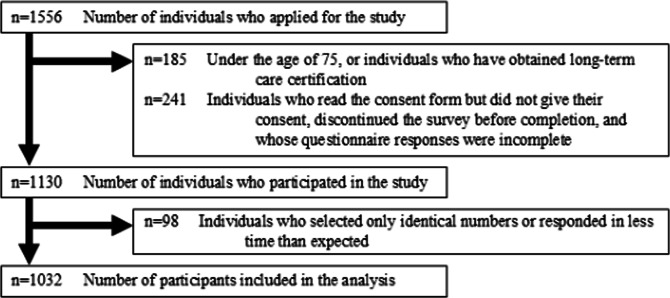
Flow of participants.

The participant flow is shown in Figure 1. Among the 1130 individuals who participated in the survey, 98 (9%) were excluded due to extremely short response times or uniform responses across all items, resulting in a final analytical sample of 1032 (1032/1130, 91.3%) participants. Table 1 presents the characteristics of participants stratified by frailty status. Using the QMCOO, 318 (30.8%) participants were classified as frail. Among the 318 participants with frailty, 218 (68.6%) reported having used at least one source of health information. The sources of health information used by older adults with frailty included television (n=173, 54.4%), the internet (n=159, 50%), health care providers (n=147, 46.2%), newspapers (n=112, 35.2%), and family members (n=73, 23%). The median (IQR) eHEALS score was 28 (IQR 24-32) among participants without frailty and 25 (IQR 21-30) among participants with frailty, indicating lower health literacy in the frail group.

**Table 1.** Participant characteristics.

	All participants (N=1032)	Nonfrail (n=714)	Frail (n=318)
		QMCOO^a^ ≤3 points	QMCOO ≥4 points
Age (years), median (IQR)	77 (75-79)	77 (75-79)	77 (76-79)
Sex, male, n (%)	518 (50.2)	339 (47.5)	179 (56.3)
Final educational attainment level, n (%)			
Up to high school	456 (44.2)	302 (42.3)	154 (48.4)
Technical or junior college	161 (15.6)	113 (15.8)	48 (15.1)
University or graduate school	413 (40)	298 (41.7)	115 (36.2)
Other	2 (0.2)	1 (0.1)	1 (0.3)
Household income^b^, n (%)			
<2 million yen	131 (12.7)	77 (10.8)	54 (17)
2–<3 million yen	178 (17.2)	116 (16.2)	62 (19.5)
3–<4 million yen	213 (20.6)	143 (20)	70 (22)
4–<5 million yen	120 (11.6)	91 (12.7)	29 (9.1)
≥5 million yen	195 (18.9)	148 (20.7)	47 (14.8)
Don’t know/prefer not to answer	195 (18.9)	139 (19.5)	56 (17.6)
Marital status, married	989 (95.8)	687 (96.2)	302 (95)
Presence of children, yes	901 (87.3)	634 (88.8)	267 (84)
Living alone, yes	187 (18.1)	120 (16.8)	67 (21.1)
Number of media sources regularly used, median (IQR)	4 (3-4)	3 (3-4)	4 (3-5)
Television, n (%)	986 (95.5)	692 (96.9)	294 (92.5)
Radio, n (%)	363 (35.2)	268 (37.5)	95 (29.9)
Internet, n (%)	904 (87.6)	634 (88.8)	270 (84.9)
Newspapers, n (%)	716 (69.4)	512 (71.7)	204 (64.2)
Magazines, n (%)	212 (20.5)	161 (22.5)	51 (16)
Books, n (%)	318 (30.8)	240 (33.6)	78 (24.5)
Accessing health information, yes, n (%)	780 (75.6)	562 (78.7)	218 (68.6)
Number of health information sources, median (IQR)	8 (3-10)	8 (4-10)	8 (0-10)
Health information sources, n (%)			
Health care providers	494 (47.9)	347 (48.6)	147 (46.2)
Family members	317 (30.7)	244 (34.2)	73 (23)
Friends/acquaintances	293 (28.4)	225 (31.5)	68 (21.4)
Neighbors	63 (6.1)	49 (6.9)	14 (4.4)
Government agencies	213 (20.6)	163 (22.8)	50 (15.7)
Long-term care and welfare professionals	45 (4.4)	33 (4.6)	12 (3.8)
Television	641 (62.1)	468 (65.5)	173 (54.4)
Radio	134 (13)	109 (15.3)	25 (7.9)
Internet	593 (57.5)	434 (60.8)	159 (50)
Newspapers	464 (45)	352 (49.3)	112 (35.2)
Magazines	100 (9.7)	73 (10.2)	27 (8.5)
Books	129 (12.5)	101 (14.1)	28 (8.8)
QMCOO score, median (IQR)	2 (1-4)	2 (1-2)	5 (4-6)
eHEALS^c^ score, median (IQR)	28 (24-31)	28 (24-32)	25 (21-30)

a QMCOO: Questionnaire of Medical Checkup for Old-Old.

b 1 million yen=US $6460.

c eHEALS: eHealth Literacy Scale.

### Association Between Health Information Sources and Frailty

Table 2 presents the results of multivariable logistic regression analyses examining the association between frailty and nonaccess to each health information source. Compared with participants without frailty, participants with frailty were less likely to access the following sources of health information: family members (OR 0.69, 95% CI 0.50‐0.95; *P*=.02), friends or acquaintances (OR 0.70, 95% CI 0.51‐0.98; *P*=.03), radio (OR 0.50, 95% CI 0.31‐0.79; *P*=.004), and newspapers (OR 0.66, 95% CI 0.50‐0.88; *P*=.005).

**Table 2.** Adjusted multivariable logistic regression analyses of the association between health information sources and frailty. The covariates included are age, sex, final educational attainment level, household income, marital status, the presence of children, living alone, and health literacy.

	Multivariable model, odds ratio (95% CI)	*P* value
Health care providers	1.08 (0.82‐1.43)	.46
Family members	0.69 (0.50‐0.95)	.02
Friends/acquaintances	0.70 (0.51‐0.98)	.03
Neighbors	0.79 (0.42‐1.49)	.67
Government agencies	0.75 (0.52‐1.07)	.14
Long-term care and welfare professionals	1.11 (0.55‐2.28)	.90
Television	0.76 (0.57‐1.00)	.06
Radio	0.50 (0.31‐0.79)	.004
Internet	0.83 (0.62‐1.11)	.46
Newspapers	0.66 (0.50‐0.88)	.005
Magazines	1.03 (0.63‐1.69)	.78
Books	0.79 (0.50‐1.26)	.29

Sex-based subgroup analyses revealed no significant interaction effects for these associations (family: *P*=.47; friends/acquaintances: *P*=.28; radio: *P*=.61; newspapers: *P*=.23), indicating that the associations were consistent across male and female participants.

## Discussion

### Novelty of the Study

To the best of our knowledge, this study is the first to examine the relationship between frailty and sources of health information among adults aged ≥75 years. Although previous studies have identified commonly accessed sources of health information among the general older population [3-5], studies focusing specifically on older adults with frailty are limited. Our findings revealed that older adults with frailty were significantly less likely than individuals without frailty to access family members, friends/acquaintances, newspapers, and radio as information sources. In addition, these associations did not differ by sex. These findings are important for understanding the extent to which older adults with frailty have difficulty accessing certain health information sources, and they provide fundamental evidence for examining methods and support strategies for delivering health information tailored to individuals with frailty by clarifying which communication channels are more susceptible to vulnerability. In particular, reduced use of interpersonal information sources, such as family and friends, may reflect weakened social connections and suggests priority issues that should be addressed in efforts to reduce information disparities and strengthen long-term care prevention policies.

### Relationship Between Health Information Sources and Frailty

#### Family and Friends/Acquaintances

Recent conceptualizations of frailty have expanded beyond physical aspects to include social dimensions. Limited interaction with family, friends, and neighbors, as well as reduced social support, are recognized components of frailty [9-11]. Older adults experiencing social isolation tend to be frail [27], and those with low social support have an elevated risk of transitioning to frailty [16]. The less frequent use of family and friends as health information sources among individuals with frailty observed in this study might reflect insufficient social support, possibly associated with frailty status.

#### Newspapers

Low health literacy has been associated with frailty [28,29], and individuals with low socioeconomic status or educational attainment tend to have a reduced ability to understand printed materials such as newspapers [30]. In this study, participants with frailty had lower eHEALS scores, household income, and final educational attainment level than individuals without frailty. Notably, the average income of older households is approximately 3 million yen (US $19,390) [24]. Our findings revealed that approximately 37% of the participants with frailty (compared with 27% of those who were not frail) are in this category. As this study was conducted online, some participants with frailty possibly accessed free news content online or refrained from subscribing to print newspapers for economic reasons. Moreover, declines in visual acuity are associated with frailty [13], and access to newspapers printed in small fonts may have been limited.

#### Radio

In rural areas of Japan, many older adults access multiple media sources such as television, radio, and newspapers, which positively influence health behaviors [6]. However, studies abroad suggest that adults aged ≥85 years tend to trust the radio less as a health information source than those aged ≤84 years. Further, listening to the radio presents two challenges: (1) the inability to ask questions to clarify information and (2) the criteria for broadcasting content are unclear, making it challenging to evaluate health information [5]. Furthermore, age-related hearing loss, which is commonly associated with frailty [31], may reduce the accessibility of audio-only media such as radio for older adults with hearing loss.

In studies targeting the general adult population and patients with cancer, sources of health information were reported as follows: television and radio (31.9%), newspapers (23.7%), the internet (54%‐56%), physicians (9%‐13%), and books (7%‐12%) [6,30]. Among these, trusted health information sources were most commonly physicians (20.4%‐53%) and health care professionals (12.2%‐60%) [17,18] suggesting that information provided by experts with medical knowledge is considered the most reliable. In this study, the sources of health information among older adults with frailty were analyzed; however, their most trusted sources were not identified. A substantial proportion of older adults with frailty in this study reported obtaining information from health care professionals. Further research is needed to identify more effective sources of health information, including those considered trustworthy.

### Strengths

A key strength of this study is its large sample size, comprising >1000 community-dwelling older adults aged ≥75 years recruited across Japan, which provides high statistical power. Moreover, sex-based subgroup analyses revealed no significant interaction effects, supporting the robustness of the main results. By targeting individuals with a higher prevalence of frailty aged ≥75 years, we successfully identified specific health information sources that older adults with frailty are less likely to access. These findings underscore the need to develop more accessible, trustworthy, and effective health information resources tailored to the needs of this population. For instance, scientifically grounded platforms operated by public or medical institutions, community-based online resources, and multimodal delivery formats combining text and audio content should be considered.

### Limitations

This study has some limitations. First, as a cross-sectional study, causal inference cannot be established. Second, participants were recruited through a web-based panel. Since older adults with frailty without internet access were likely excluded, sampling bias may have occurred, restricting the generalizability of the findings to relatively healthier, internet-using older adults in Japan. Consequently, the results of this study may underestimate disparities in access to health information among older adults with frailty. In contrast, although the prevalence of frailty among older adults in Japan is reported to be 7.4% [8], the proportion of older adults with frailty identified in this study was 30.8%. This suggests that many older adults who are often excluded from conventional surveys—such as those with difficulty walking, those who tend to remain indoors, and those with limited social interaction and an increased risk of frailty—may have been included in this study. Third, frailty was not assessed using objective measures such as gait speed or grip strength, which may have reduced the accuracy of the assessment. Finally, confounding factors related to health status and cognitive function, such as the frequency and content of access to health information, severe cognitive impairment, major comorbidities, physical functional limitations, and mobility, were not adjusted for.

### Conclusions

Our findings suggest that older adults with frailty were less likely to obtain health information from interpersonal sources such as family and friends, as well as from traditional media such as radio and newspapers, than individuals without frailty. These findings are valuable for developing effective and personalized strategies to deliver health information to older adults with frailty.

## Supplementary material

10.2196/83642 Multimedia Appendix 1 Items and response options of the Questionnaire for Medical Checkup of Old-Old and eHealth Literacy Scale.

## Abbreviations

eHEALS: eHealth Literacy Scale
OR: odds ratio
QMCOO: Questionnaire for Medical Checkup of Old-Old
