# Supplementary material for: Association Between Access to Health Information and Frailty in Older Japanese Adults: Web-Based Cross-Sectional Study
Source: Online J Public Health Inform. 2026 Feb 27;18:e83642. doi: 10.2196/83642 (PMC12954718; doi:10.2196/83642)
Supplement: Multimedia Appendix 1 [file ojphi-v18-e83642-s001.docx]

Supplementary table 1. Proportion of each response to the Questionnaire for Medical Checkup of Old-Old

| Items | Answers |
| --- | --- |
| 1. How is your health condition? | Excellent |
|  | Good |
|  | Fair |
|  | Poor^a^ |
|  | Very poor^a^ |
| 2. Are you satisfied with your daily life? | Satisfied |
|  | Moderately satisfied |
|  | Moderately dissatisfied^a^ |
|  | Dissatisfied^a^ |
| 3. Do you eat three times a day? | Yes |
|  | No^a^ |
| 4. Do you have any difficulties eating tough foods compared to 6 months ago? | Yes^a^ |
|  | No |
| 5. Have you choked on your tea or soup recently? | Yes^a^ |
|  | No |
| 6. Have you lost 2 kg or more in the past 6 months? | Yes^a^ |
|  | No |
| 7. Do you think you walk slower than before? | Yes^a^ |
|  | No |
| 8. Have you experienced a fall in the past year? | Yes^a^ |
|  | No |
| 9. Do you go for a walk for your health at least once a week? | Yes |
|  | No^a^ |
| 10. Do your family or friends point out your memory loss?  (e.g., “You ask the same question over and over again.”) | Yes^a^ |
|  | No |
| 11. Do you find yourself not knowing today's date? | Yes^a^ |
|  | No |
| 12. Do you smoke? | Yes^a^ |
|  | No |
|  | I quit |
| 13. Do you go out at least once a week? | Yes |
|  | No^a^ |
| 14. Do you keep up regular communication with your family and friends? | Yes |
|  | No^a^ |
| 15. When you are not feeling well, do you have anyone you can talk to? | Yes |
|  | No^a^ |

a: These answers are used to calculate points for each question, and the scores range from 0 to 15.

Supplementary table 2. Proportion of answers of eHEALTH

| Items | Answers |
| --- | --- |
| 1. I know how to find helpful health resources on the Internet | 1 = strongly disagree  2 = disagree  3 = undecided  4 = agree  5 = strongly agree |
| 2. I know how to use the Internet to answer my health questions |  |
| 3. I know what health resources are available on the Internet |  |
| 4. I know where to find helpful health resources on the Internet |  |
| 5. I know how to use the health information I find on the Internet to help me |  |
| 6. I have the skills I need to evaluate the health resources I find on the Internet |  |
| 7. I can tell high quality from low quality health resources on the Internet |  |
| 8. I feel confident in using information from the Internet to make health decisions |  |
